# Morphological and molecular evidence for cryptic species of springsnails [genus *Pseudamnicola* ( *Corrosella*) (Mollusca, Caenogastropoda, Hydrobiidae)]

**DOI:** 10.3897/zookeys.190.2555

**Published:** 2012-05-04

**Authors:** Diana Delicado, Marian A. Ramos

**Affiliations:** 1 Museo Nacional de Ciencias Naturales (CSIC), José Gutiérrez Abascal 2, 28006-Madrid, Spain

**Keywords:** Hydrobiidae, *Pseudamnicola (Corrosella)*, *Pseudamnicola (Corrosella) astieri* (Dupuy 1851), *Pseudamnicola (Corrosella) hauffei* sp. n., France, Spain, Iberian Peninsula, taxonomy, COI, cryptic species, conservation

## Abstract

Several *Pseudamnicola (Corrosella)* populations of the central and eastern Iberian Peninsula have been ascribed to *Pseudamnicola (Corrosella) astieri* (Dupuy, 1851), though recent evidence demonstrates the species could be endemic to the departments of Var and Alpes-Maritimes in France. Through the identification of cryptic species using a combined morphological and phylogenetic approach, this paper provides a detailed morphological description of *Pseudamnicola (Corrosella) astieri*, clarifying its taxonomic boundaries and confirming it as a French endemic. In parallel, by comparing *Pseudamnicola (Corrosella)* populations from the provinces of Castellón and Valencia in Eastern Spain, it was observed that rather than *Pseudamnicola (Corrosella) astieri* they represented a new species here described as *Pseudamnicola (Corrosella) hauffei* **sp. n.** Among other characters, the two species show marked differences in shell shape, male and female genital systems, radular formula and concentration of the nervous system. *Pseudamnicola (Corrosella) hauffei* **sp. n.** was also compared morphologically to another two *Pseudamnicola (Corrosella)* species living in nearby areas [*Pseudamnicola (Corrosella) hinzi* Boeters, 1986 and *Pseudamnicola (Corrosella) navasiana* (Fagot, 1907)], molecularly to *Pseudamnicola (Corrosella) falkneri* (Boeters, 1970), the type species of the subgenus, and to the rest of the *Pseudamnicola (Corrosella)* species described so far. Morphological differentiation between the species is supported by a genetic divergence of 7.4% inferred from a partial sequence (658 bp) of the mitochondrial gene cytochrome c oxidase subunit I (COI). On the basis of an average 8% (5.39 to 11.15%) divergence estimated for the COI gene in other *Pseudamnicola (Corrosella*) species reported in GenBank, the existence of two specific entities is here proposed, which will have impact on conservation policies both in France and in Spain.

## Introduction

The Mediterranean basin, and within it the Iberian Peninsula, has been identified as a biodiversity hotspot for animal species including those of hydrobiid gastropods (Arconada and Ramos 2003). The Peninsula’s hydrobiid fauna comprises a large number of endemic genera and species with restricted distribution areas, in addition to those showing a typically circummediterranean distribution. Many hydrobiid species and populations are threatened, and in some cases, in danger of extinction due to the fragile nature of the ecosystems they inhabit. The freshwater genus *Pseudamnicola* Paulucci, 1878, among the most cumbersome in terms of taxonomy, is one of the largest and most diverse groups of Hydrobiidae Stimpson, 1965, with around 85 nominal species (Boeters 1976, Ghamizi et al. 1997, Glöer et al. 2010, Glöer and Pesič 2009, Fauna Europaea 2011). However, many of these taxa require confirmation of their taxonomic status since they have yet to be morphologically well characterized. Delimiting species is essential both to assess diversity and to inform conservation agencies about possible strategies to preserve this sensitive group of molluscs and their habitats. For some groups of hydrobiids of similar morphology, small size and simple shells and anatomy, describing species boundaries is particularly challenging since most diagnostic characters are related to the morphometrics of soft parts. Recent papers have demonstrated that molecular data are useful to support the morphological delimitation of hydrobiid genera and species (Hershler et at. 2003, Szarowska et al. 2005, Arconada and Ramos 2006, Arconada et al. 2007) and that a combined approach using morphological and molecular data can help reveal intraspecific variability unveiling cryptic species within the genus *Pseudamnicola* (Szarowska et al. 2006, Delicado et al. 2012).

Two subgenera are currently recognized within the *Pseudamnicola*: *Pseudamnicola (Corrosella*), occurring in the Iberian Peninsula and one small area in the South of France; and *Pseudamnicola (Pseudamnicola)*, widely distributed in freshwater ecosystems of the Mediterranean basin. The diversity of the subgenus *Corrosella* is much lower than that of *Pseudamnicola* and only 11 nominal species (described by: Dupuy 1851, Fagot 1907, Boeters 1970, 1984, 1986, 1988, 1999, Girardi 2009 and Delicado et al. 2012) have been ascribed to this subgenus in a more restricted distribution area.

One of these 11 species is *Pseudamnicola (Corrosella) astieri* (Dupuy, 1851), originally described from the surroundings of Grasse in the department of Alpes-Maritimes (France). Several other species were later cited from the neighbouring Var department (*Bythinella anteisensis* Berenguier 1882, *Bythinella berenguieri* Bourguignat in Berenguier 1882, *Bythinella doumeti* Bourguignat in Locard 1893, among others) and synonymised with *Pseudamnicola (Corrosella) astieri* (see Falkner et al. 2002 and Girardi 2009 for a review). Then, when *Corrosella* Boeters, 1970 was introduced, *Bythinella anteisensis* was included by the author and *Bythinella berenguieri* considered a younger synonym. In 1981, Gasull recorded the presence of *Pseudamnicola (Corrosella) astieri* (Dupuy) in the Castellón province (Spain). However, Falkner et al. (2002) later claimed certain misunderstandings in the exchange of information between Boeters and Gasull (Boeters pers. com.) which had led to the report that *Pseudamnicola (Corrosella) astieri* also inhabited several central and eastern Spanish provinces (see Gasull 1981, Vidal-Abarca and Suárez 1985). As a result of Falkner’s review, the Catalogue of Continental Molluscs in France (Falkner et al. 2002) included this entity as an endemism of the Var department.

Our paper provides a wide conchological and anatomical description of a new species of *Pseudamnicola (Corrosella)* from eastern Spain (Iberian Peninsula), *Pseudamnicola (Corrosella) hauffei* sp. n., and, through its re-description, compares it with the species *Pseudamnicola (Corrosella) astieri* from Var (France)and with other *Pseudamnicola (Corrosella)* species with close-by distribution areas in the Iberian Peninsula. Morphological studies were combined with cytochrome *c* oxidase subunit I (COI) sequence analysis in the light of previously published molecular data (Delicado et al. 2012) to test divergence and phylogenetic relationships among *Pseudamnicola (Corrosella)* species. Overall, our results delimit the two species indicating that *Pseudamnicola (Corrosella) astieri* is an endemic species of the Var department of France and add a new clade to the already known phylogeny of the subgenus *Corrosella*. These results would necessarily have to be considered to design the conservation strategies for these restricted species both in France and in Spain.

## Material and methods

**Figure 1.**
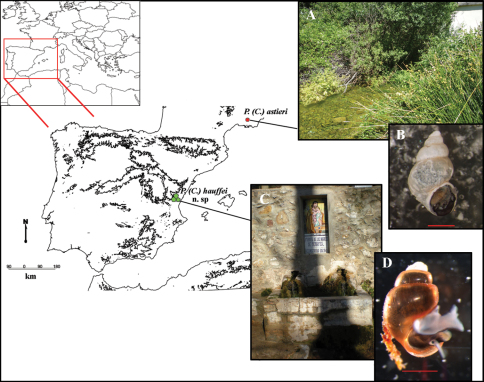
Map of localities of *Pseudamnicola (Corrosella) astieri* and *Pseudamnicola (Corrosella) hauffei* sp. n. **A** Photograph of Source d’Argens, Brue-Aurillac, Var, France **B** Preserved specimen of *Pseudamnicola (Corrosella) astieri* **C** Photograph of Nogales spring, Benafer, Castellón, Spain (type locality of *Pseudamnicola (Corrosella) hauffei* sp. n.) **D** Alive specimen of *Pseudamnicola (Corrosella) hauffei* sp. n. Scale bar in B and D represents 1 mm.

The study area comprised the Departments of Alpes-Maritimes and Var in southeastern France and the provinces of Castellón and Valencia in eastern Spain. Specimens were collected from several sites in this area (see Figure 1) and deposited in the Collection of Molluscs of the Museo Nacional de Ciencias Naturales (MNCN), Madrid, Spain.

Anatomical observations and morphometric measurements were made on specimens relaxed with menthol crystals and fixed in ethanol following the procedures described in Ramos et al. (2000) and Arconada and Ramos (2001). Morphological descriptions are based on the terminology of Hershler and Ponder (1998) except for characters not described or included in that paper for which we use the nomenclature of Delicado et al. (2012). Spire whorls were counted following the method of Ramos et al. (2000). The number of specimens undergoing morphometry, the localities, and sampling dates for each species are indicated in the corresponding section of the text and tables. The unequal sample size of measured specimens affects both standard deviation and coefficient of variation of the morphometric study. In order to correct these biases, we followed the methods of Holtzman (1950) and Biemann and Kearney (2010) respectively. All calculations have been done using the package MBESS (Kelley and Lai 2011) for the R statistical environment (R Development Core Team 2011). Student´s t-test was used to check statistical differences between shell dimensions in both species.

Specimens were dissected under a Leica MZ 16 A stereomicroscope and photographed using a Nikon ds fi1 camera. All measurements were made using Nis-Elements V. 2.2. software. Anatomical illustrations were prepared from camera lucida drawings. Environmental scanning electron microscope (ESEM) images of shells were captured using a Philips Quanta 200 in low-vacuum mode, after removal of the periostracum by immersion in 5% sodium hypochlorite and then cleaning by ultrasonication. The radula and operculum were cleaned by immersion in KOH solution (10g/l) at room temperature. Both structures were then rinsed in distilled water and air-dried before mounting on stubs and coating with a thin (10–20 nm) gold layer in an Emitech K550X sputter coating unit followed by observation in high-vacuum mode.

Total DNA was isolated from the foot tissue of the snails using the ChargeSwitch gDNA Micro Tissue (Invitrogen, Paisley, UK) extraction kit. Partial COI sequences were amplified by polymerase chain reaction (PCR) using LCO1490 (Folmer et al. 1994) and COR722 (Davis et al. 1998) as primers, following the protocol described in Delicado et al. 2012. We examined three specimens from the type locality of *Pseudamnicola (Corrosella) hauffei* and three specimens from the Source d’Argens of *Pseudamnicola (Corrosella) astieri* and the sequences obtained were edited using the SEQUENCHER v.4.6 program (Gene Code Corporation, Ann Arbor, MI, USA). A molecular data set was created together with other published sequences for *Pseudamnicola* (see Table 1 for Genbank accession numbers). Uncorrected divergences were calculated in PAUP 4b10 and Bayesian analysis was performed with MRBAYES 3.1.2 (Huelsenbeck 2000; Huelsenbeck and Ronquist 2001) employing two parallel runs of 5 million of generations and sampling one every 1000 replicates. The 10% of sampled trees were discarded as burn-in (see details in Delicado et al. 2012).

**Table 1.** Species name, locality details, Genbank accession numbers and publication references for mtCOI sequences.

Species name	Locality	Genbank accession number	Reference
*Pseudamnicola (Corrosella) luisi*	La Gitana spring, La Peza, Granada, Spain.	JF312220	Delicado et al. 2012
*Pseudamnicola (Corrosella) falkneri*	La Armada spring, Orce, Granada, Spain.	JF312224	Delicado et al. 2012
*Pseudamnicola (Corrosella) manueli*	La Garganta stream, Nava de San Pedro, Jaén, Spain.	JF312227<br/> JF312228	Delicado et al. 2012
*Pseudamnicola (Corrosella) bareai*	Spring in Ermita de las Santas, Granada, Spain.	JF312225<br/> JF312226	Delicado et al. 2012
*Pseudamnicola (Corrosella) marisolae*	Pilar del Mono spring, Dúrcal, Granada, Spain.	JF312218<br/> JF312219	Delicado et al. 2012
*Pseudamnicola (Corrosella) iruritai*	Don Pedro spring, Loja, Granada, Spain.	JF312221<br/> JF312222	Delicado et al. 2012
*Pseudamnicola (Corrosella) andalusica*	La Salud spring, Toscarejo, Jaén, Spain.	JF312223	Delicado et al. 2012
*Pseudamnicola (Pseudamnicola) lucensis*	Thermal spring in Bagni di Lucca, Tuscany, Italy.	AF367651	Wilke et al. 2001
*Pseudamnicola (Pseudamnicola) macrostoma negropontina*	Artificial pond in Marmaris, Evvoia island, Greece.	EF061915	Szarowska et al. 2006
*Hydrobia acuta acuta*	Lac de Tunis, Tunisia.	AF278804	Wilke et al. 2000
*Pyrgula annulata*	Lake Garda, Brescia, Italy.	AY341258	Szarowska et al. 2005

### Abbreviations used in the text and tables

*Shell and operculum characters*:AH: aperture height; AL: aperture length; AW: aperture width; LBW: length of body whorl; NL: length of opercular nucleus; NW: width of opercular nucleus; NSW: number of spire whorls; OL: operculum length; OLWL: length of the last whorl of the operculum; OLWW: width of the last whorl of the operculum; OW: operculum width; SL: shell length; SW: shell width; WAW: width of the antepenultimate whorl; WBW: width of the body whorl; WPW: width of the penultimate whorl.

*Anatomical characters*: Ag: albumen gland; Bc: bursa copulatrix; CC: cerebral commissure; Cg: capsule gland; Ct: ctenidium; dBc: duct of the bursa copulatrix; LCG: left cerebral ganglion; LPG: left pleural ganglion; Os: osphradium; P: penis; Po: pallial oviduct; Pr: prostate gland; RCG: right cerebral ganglion; Ro: renal oviduct; RPG: right pleural ganglion; SR: seminal receptacle; Ss: style sac; St: stomach; SubC: suboesophageal connective; SubG: suboesophageal ganglion; SupC: supraoesophageal connective; SupG: supraoesophageal ganglion; L: length; W: width. The concentration of the nervous system was measured as the “RPG” ratio (Davis et al. 1976) and also characterised using the categories of Davis et al. (1984, 1986, 1992) as follows: dorsal nerve ring concentrated (≤ 0.29); moderately concentrated (0.30–0.49); elongated (0.50–0.67); extremely elongated (≥ 0.68).

*Collections*.BOE, Boeters, München, Bundesrepublik Deutschland; MHNG, Muséum d’histoire naturelle de la Ville de Genève, Switzerland; MNCN, Museo Nacional de Ciencias Naturales, Madrid, Spain.

*Collectors*. B.A., B. Arconada; C.N., C. Noreña; D.D., D. Delicado; D.M., D. Moreno; J.M.R, J.M. Remón; R.A., R. Araujo.

## Results

### Systematic descriptions

#### Pseudamnicola (Corrosella) astieri

(Dupuy, 1851)

http://species-id.net/wiki/Pseudamnicola_astieri

Figs 2 [Fig F3] –4

Hydrobia astierii Dupuy, 1851: 556–557, pl. XXVII, fig. 12, Paris (Type loc. surroundings of Grasse, Alpes-Maritimes, France [shell description]).
Paludinella astieri (Dupuy): Frauenfeld 1865: 575.
Bythinella astieri (Dupuy): Locard 1882: 227; Bérenguier 1882: 83; Locard 1893: 79, fig. 81 (shell description); Berenguier 1902: 378, pl. 16 fig. 6 (1990).
Bythinella anteisensis Bérenguier, 1882: 83, 89–90 (Type loc. Foux de Draguignant, Var, France [shell description]); Bérenguier 1902: 378–379 (shell description) pl. 16 fig. 7 (1990). (Synonymy: Girardi 2009: 56).
Bythinella berenguieri Bourguignat in Bérenguier, 1882: 83, 99–100 (Type loc. Foux de Draguignant, Var, France [shell]); Bérenguier 1902: 379–380 (shell description) pl. 16 fig. 8 (1990) (Synonymy: Girardi 2009: 56).
Bythinella doumeti Bourguignat in Locard, 1893: 91. (Type loc. surroundings of Nimes, Gard, France [shell description]) (Synonymy: Falkner et al. 2002: 81, after revision of two syntypes in the Bourguignat collection, MHNG).
Corrosella anteisensis (Bérenguier): Boeters, 1970: 64, figs. 2, 4, 7, 9 [(shell, operculum, male and female genital systems of topotypes; Boeters could not find the syntypes)], (=*Bythinella berenguieri* Bourguignat in Bérenguier). (Synonymy: Girardi 2009: 56).
Pseudamnicola (Corrosella) astierii (Dupuy): Falkner et al. 2002: 29, 80-81;Girardi 2009: 56–61, figs. 1–3 (Var, France: Source d’Argens, Source du Pavillon, Source de la Foux à Draguignan [shell and anatomy]).

##### Type locality.

Surroundings of Grasse, France (Dupuy, 1851).

##### Type material.

Boeters (1970) reported the existence of one specimen with the label “*Paludinella astieri*, typus ex Dupuy” in Paladilhe’s collection at the Faculté des Sciences, Montpellier, France. We tried in vain to confirm the existence of such material at the university mentioned. Consequently, we should consider that the type specimen is presently inaccessible for study. However, some topotypes of *Corrosella anteisensis* (Bérenguier) from Foux à Draguignan, Var exist: BOE 261, 285 a-c, 291b Boeters (1970) and Girardi (2009). This author also reported *Pseudamnicola (Corrosella) astieri* from Source d’Argens, Brue-Aurillac à Seillons, Var and the Source du Pavillon, Ruisseau Fauvery à Pontevès, Var (Girardi 2009).

**Figure 2.**
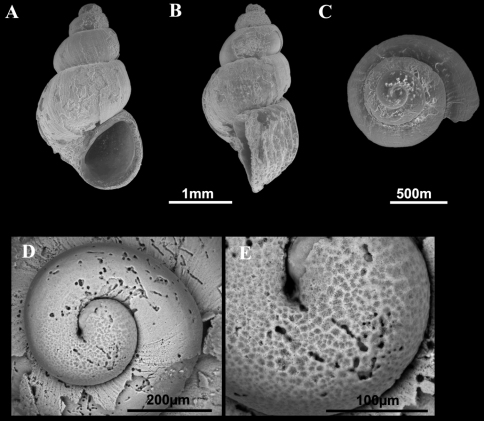
Shells of *Pseudamnicola (Corrosella) astieri*. **A–E** Shells from Source d’Argens, Brue-Aurillac, Var, France **D**–**E** Protoconch and detail of the microsculpture from the protoconch.

**Figure 3. F3:**
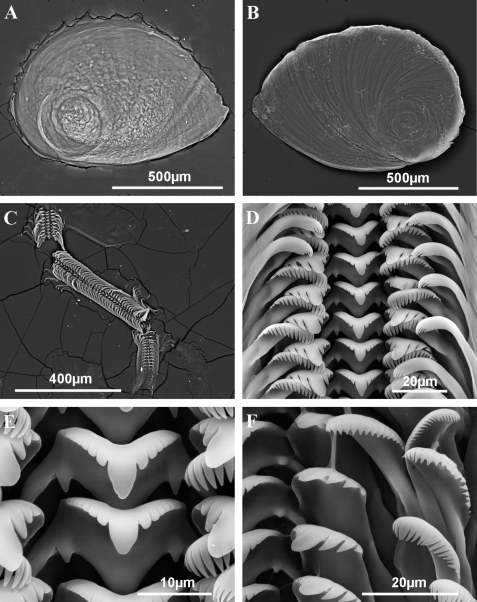
Operculum and radula of *Pseudamnicola (Corrosella) astieri* from Source d’Argens, Brue-Aurillac, Var, France. **A** Internal side of the operculum **B** External side of the operculum **C** Radula **D** Rows of teeth of the radula **E** Central tooth **F** Lateral, internal and external marginal teeth.

**Figure 4.**
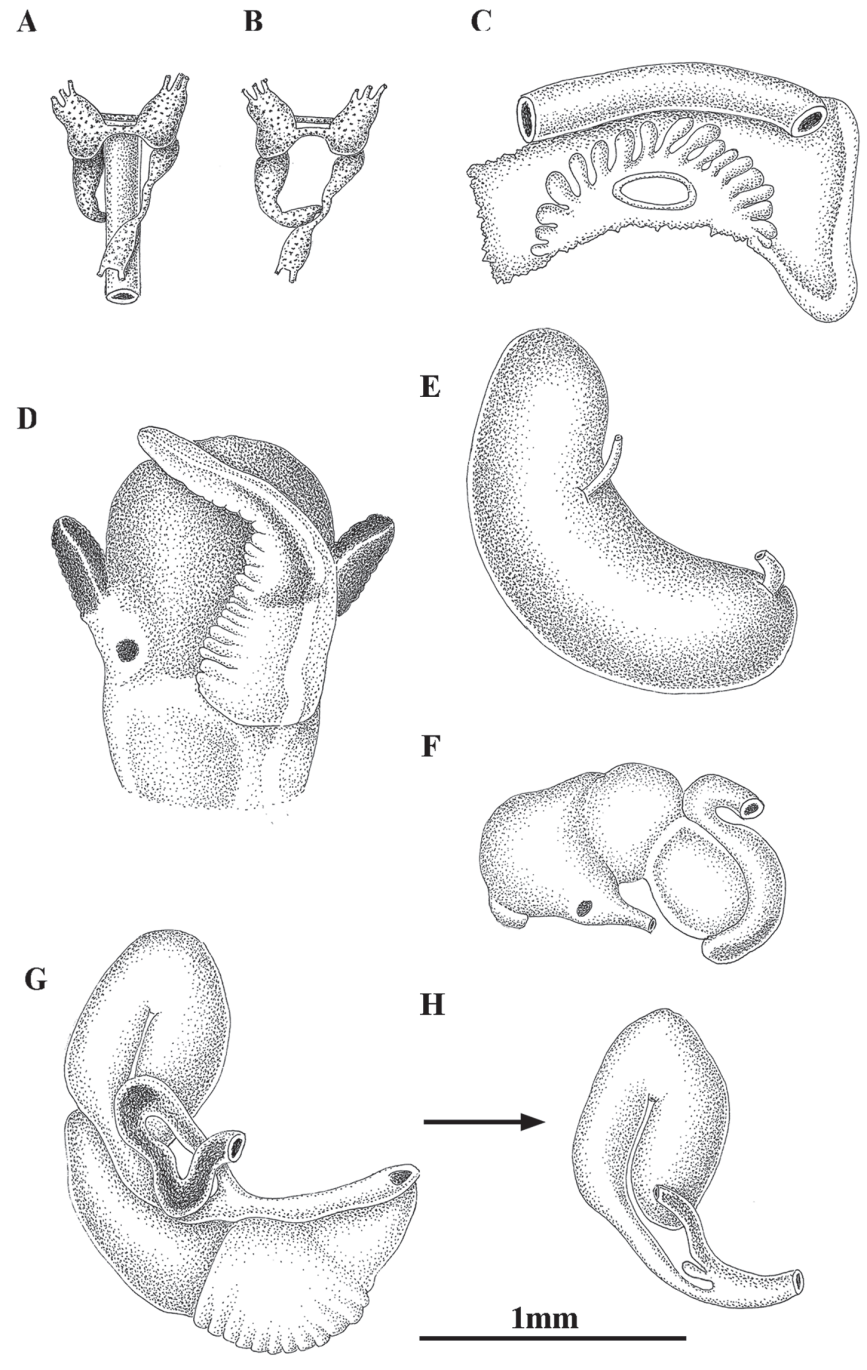
Anatomy of *Pseudamnicola (Corrosella) astieri* from Source d’Argens, Brue-Aurillac, Var, France. **A, B** Partial nervous system **C** Ctenidium and osphradium **D** Head of a male and penis **E** Prostate gland **F** Stomach **G** Female genitalia **H** Bursa copulatrix and seminal receptacle.

##### Material examined.

A few specimens collected from Source d’Argens , Brue-Aurillac, Var, France after finding the type area and other localities in Alpes-Maritimes and Var practically destroyed by severe storms. A total of two females and four males have been examined for anatomical descriptions.

##### Localities.

Source d’Argens, Brue-Aurillac, Var, France, 43°30.24'N, 5°54.43'E, D.D., 21 June 2010, MNCN 15.05/60025 (70° ethanol, Figures 2–4) and MNCN/ADN 54949–54951 (absolute ethanol). For more localities, see Girardi 2009.

##### New diagnosis.

Shell yellowish or whitish with a body whorl occupying 2/3 shell length and a deep suture between whorls; protoconch microsculpture granulated; central radular tooth formula 7-C-7; style sac surrounded by a black pigmented intestine; elongate bursa copulatrix U-shaped; elongate seminal receptacle without duct; penis slender with a black patch of pigmentation and some folds in its middle region; nervous system brown pigmented with supraoesophageal connective about two times longer than suboesophageal.

##### Description.

Shellovate-conic with 4–4.75 spire whorls, height 2.5–3.5 mm (Figure 2A–C; Table 2); periostracum yellowish or whitish; protoconch approximately 370 µm wide with 1.5 whorls and a nucleus around 150 µm long (Figure 2D, E); protoconch microsculpture granulated, more intense on apex (Figure 2E); body whorl about 2/3 total length; teleoconch whorls convex with a deep suture; peristome orthocline; aperture complete, oval, with an inner lip thicker than outer lip; peristome margin simple, straight (Figure 2B).

**Table 2.** Shell measurements (in mm) of *Pseudamnicola (Corrosella) astieri* from d’Argens spring, Seillons, France and *Pseudamnicola (Corrosella) hauffei* sp. n. from Los Nogales spring, Benafer, Castellón, Spain. Probability *p* values of *T*-test are provided for each variable, n.s. = no significant.

	*Pseudamnicola (Corrosella) astieri*<br/> (n=11)			*Pseudamnicola (Corrosella) hauffei* sp. n.<br/> (n=18)			T-test<br/> (*p* values)
	**Mean<br/> (Max-Min)**	**S_D_N**	**CV**	**Mean<br/> (Max-Min)**	**S_D_N**	**CV**	
SL	3.11<br/> (3.56–2.58)	0.25	0.08	2.50<br/> (2.85–2.17)	0.18	0.07	p < 0.001
SW	1.92<br/> (2.28–1.74)	0.15	0.08	1.59<br/> (1.78–1.44)	0.10	0.06	p < 0.001
SL/SW	1.62<br/> (1.76–1.45)	0.10	0.06	1.57<br/> (1.73–1.46)	0.07	0.04	n.s.
AH	1.37<br/> (1.78–1.26)	0.14	0.10	1.20<br/> (1.36–1.04)	0.08	0.07	p < 0.001
SL–LBW	1.10<br/> (1.39–0.76)	0.16	0.15	0.69<br/> (1.00–0.49)	0.13	0.19	p < 0.001
WBW	1.76<br/> (1.89–1.59)	0.09	0.05	1.42<br/> (1.57–1.29)	0.07	0.05	p < 0.001
AL	1.39<br/> (1.72–1.28)	0.12	0.08	1.26<br/> (1.41–1.11)	0.09	0.07	p < 0.01
AW	1.01<br/> (1.33–0.85)	0.14	0.14	0.89<br/> (1.11–0.78)	0.08	0.09	p < 0.01
WPW	1.23<br/> (1.33–1.11)	0.08	0.07	0.95<br/> (1.12–0.80)	0.08	0.08	p < 0.001
WAW	0.19<br/> (0.32–0.15)	0.06	0.32	0.28<br/> (0.37–0.19)	0.04	0.14	p < 0.001
NSW	4.30<br/> (4.75–4.00)	0.31	0.07	4.18<br/> (4.50–4.00)	0.21	0.05	n.s.

SD_N_, Unbiased estimate for Standard Deviation, CV, Coefficient of Variation.

Operculum corneous, yellowish, thin, pliable, ellipsoidal, paucispiral with nucleus submarginal (Figure 3A, B; Table 3); muscle attachment area oval, located near the nucleus.

**Table 3.** Operculum measurements (in mm) of *Pseudamnicola (Corrosella) astieri* from d’Argens spring, Seillons, France and *Pseudamnicola (Corrosella) hauffei* sp. n. from Los Nogales spring, Benafer, Castellón, Spain.

	*Pseudamnicola (Corrosella) astieri* (n=5)			*Pseudamnicola (Corrosella) hauffei* sp. n. (n=7)		
	Mean (Max-Min)	S_D_N	CV	Mean (Max-Min)	S_D_N	CV
OL	1.10 (1.18–0.96)	0.05	0.05	1.08 (1.18–0.96)	0.08	0.08
OW	0.79 (0.84–0.73)	0.04	0.05	0.74 (0.84–0.66)	0.06	0.08
OLWL	0.48 (0.56–0.40)	0.05	0.11	0.49 (0.63–0.36)	0.08	0.17
OLWW	0.34 (0.40–0.25)	0.05	0.16	0.33 (0.38–0.29)	0.03	0.09
NL	0.47 (0.52–0.39)	0.04	0.09	0.36 (0.44–0.26)	0.06	0.17
NW	0.36 (0.42–0.31)	0.04	0.12	0.29 (0.45–0.21)	0.06	0.22

SD_N_, Unbiased estimate for Standard Deviation, CV, Coefficient of Variation.

Radula intermediate length (20% total shell length) bearing some 50 rows of teeth (Figure 3C, Table 4); central tooth has a tongue-shaped median cusp and seven blunt lateral cusps (Figure 3D, E); lateral teeth with three tapered cusps on each side of a long central tongue-shaped cusp; inner marginal teeth have 18 sharp cusps, shortening towards the tooth base; outer marginal teeth with 19 sharp cusps (Figure 3D, F).

**Table 4.** Radula formulae and measurements (in mm) of three radulae of *Pseudamnicola (Corrosella) astieri* from d’Argens spring, Seillons, France and three of *Pseudamnicola (Corrosella) hauffei* sp. n. from Los Nogales spring, Benafer, Castellón, Spain.

	*Pseudamnicola (Corrosella) astieri*	*Pseudamnicola (Corrosella) hauffei* sp. n.
Central teeth	7+C+7/1–1	5+C+5/1–1
Central teeth width	~ 20 µm	~ 15 µm
Lateral teeth	3–C–3	3–C–3
Inner marginal teeth	≥ 18 cusps	≥ 15 cusps
Outer marginal teeth	≥ 19 cusps	≥ 19 cusps
Radula length	~ 700 µm	~ 600 µm
Radula width	~ 90 µm	~ 95 µm
Number of rows	~ 55	~ 50

*Pigmentation and anatomy*: Head dark brown pigmented from snout to neck (Figure 4D); pigmentation clearer on neck; tentacles also brown pigmented except for a narrow band on these and on ocular lobes; snout long as wide, with medial lobation; foot intermediate length and pigmented in dorsal region. Ctenidium in middle region of pallial cavity filling ca. 70% of its length with 17–18 gill filaments; osphradium intermediate width under central gill filaments (Figure 4C, Table 5). Stomach slightly longer than wide with a small posterior caecum; style sac shorter than stomach and surrounded by intestine black pigmented (Figure 4F, Table 5).

**Table 5.** Ctenidium, osphradium and digestive system measurements (in mm) of *Pseudamnicola (Corrosella) astieri* from d’Argens spring, Seillons, France and *Pseudamnicola (Corrosella) hauffei* sp. n. from Los Nogales spring, Benafer, Castellón, Spain.

	*Pseudamnicola (Corrosella) astieri* <br/> (n=5)			*Pseudamnicola (Corrosella) hauffei* sp. n.<br/> (n=7)		
	**Mean<br/> (Max-Min)**	**S_D_N**	**CV**	**Mean<br/> (Max-Min)**	**S_D_N**	**CV**
Ct L	1.06<br/> (1.25–0.90)	0.15	0.14	1.11<br/> (1.27–0.92)	0.16	0.14
Os L	0.32<br/> (0.43–0.20)	0.09	0.27	0.33<br/> (0.45–0.24)	0.07	0.22
Os W	0.13<br/> (0.15–0.11)	0.02	0.16	0.21<br/> (0.25–0.15)	0.03	0.15
Ss L	0.59<br/> (0.67–0.50)	0.06	0.11	0.62<br/> (0.66–0.58)	0.03	0.05
Ss W	0.35<br/> (0.37–0.31)	0.02	0.06	0.37<br/> (0.41–0.33)	0.03	0.08
St L	0.71<br/> (0.74–0.66)	0.04	0.06	0.73<br/> (0.84–0.66)	0.06	0.09
St W	0.61<br/> (0.67–0.56)	0.04	0.07	0.69<br/> (0.78–0.61)	0.06	0.09

SD_N_, Unbiased estimate for Standard Deviation, CV, Coefficient of Variation.

Female genitalia with a slender pallial oviduct (Figure 4G; Table 6); capsule gland longer than albumen gland and consisting of two regions, the posterior one being more transparent; elongate bursa copulatrix, long, folded and U-shaped with a duct about 70% of bursa length; renal oviduct straight and less pigmented from the insertion point of the bursal duct to where it begins to fold and black pigmented making one or two loops; elongate seminal receptacle without duct (Figure 4H) joining renal oviduct just before the point where the bursal duct joins the renal oviduct.

**Table 6.** Female and male genitalia measurements (in mm) of two females and four males of *Pseudamnicola (Corrosella) astieri* from d’Argens spring, Seillons, France and four females and four males of *Pseudamnicola (Corrosella) hauffei* sp. n. from Los Nogales spring, Benafer, Castellón, Spain.

	*Pseudamnicola (Corrosella) astieri*			*Pseudamnicola (Corrosella) hauffei* sp. n.		
	**Mean<br/> (Max-Min)**	**S_D_N**	**CV**	**Mean<br/> (Max-Min)**	**S_D_N**	**CV**
Po L	2.22<br/> (2.35–2.08)	0.24	0.11	2.04<br/> (2.32–1.75)	0.26	0.13
Po W	0.58<br/> (0.59–0.56)	0.03	0.04	0.45<br/> (0.49–0.41)	0.04	0.10
Ag. L	0.95<br/> (1.04–0.84)	0.18	0.18	0.81<br/> (1.00–0.71)	0.14	0.17
Cg. L	1.01<br/> (1.11–0.91)	0.18	0.17	1.00<br/> (1.11–0.93)	0.09	0.09
SR1 L	0.14<br/> (0.15–0.14)	0.01	0.09	0.18<br/> (0.22–0.15)	0.03	0.18
BC L	1.24<br/> (1.32–1.15)	0.15	0.12	1.37<br/> (1.56–0.95)	0.30	0.22
BC W	0.30<br/> (0.35–0.25)	0.09	0.29	0.31<br/> (0.36–0.25)	0.05	0.18
dBC L	0.60<br/> (0.75–0.65)	0.09	0.15	0.62<br/> (0.72–0.46)	0.13	0.21
Pr L	1.67<br/> (1.86–1.55)	0.14	0.08	1.37<br/> (1.46–1.26)	0.09	0.06
Pr W	0.58<br/> (0.69–0.52)	0.09	0.15	0.45<br/> (0.51–0.41)	0.05	0.12
P L	1.26<br/> (1.35–1.15)	0.09	0.07	1.28<br/> (1.60–1.12)	0.24	0.19
P W	0.37<br/> (0.40–0.33)	0.03	0.09	0.66<br/> (0.75–0.50)	0.12	0.18
PL/Head length	1.02<br/> (1.16–0.86)	0.16	0.16	0.91<br/> (1.07–0.82)	0.15	0.17

SD_N_, Unbiased estimate for Standard Deviation, CV, Coefficient of Variation.

Male genitalia bear a bean-shaped prostate gland about three times longer than wide (Figure 4E, Table 6); penis long, slender, with a black patch of pigmentation and some folds in its middle region; attachment area behind right eye (Figure 4D); penial duct scarcely visible running straight close to the outer penis margin.

Nervous system brown pigmented, consisting of disperse points of pigmentation; cerebral ganglia equal in size; supraoesophageal connective more than two times longer than suboesophageal (Figure 4A, B; Table 7). Mean RPG ratio 0.42 (moderately concentrated).

**Table 7.** Nervous system measurements (in mm) of *Pseudamnicola (Corrosella) astieri* from d’Argens spring, Seillons, France and *Pseudamnicola (Corrosella) hauffei* sp. n. from Los Nogales spring, Benafer, Castellón, Spain.

	*Pseudamnicola (Corrosella) astieri*<br/> (n=5)			*Pseudamnicola (Corrosella) hauffei* sp. n.<br/> (n=5)		
	**Mean<br/> (Max-Min)**	**S_D_N**	**CV**	**Mean<br/> (Max-Min)**	**S_D_N**	**CV**
LRCG	0.22<br/> (0.24–0.20)	0.02	0.10	0.21<br/> (0.23–0.18)	0.02	0.10
LLCG	0.22<br/> (0.25–0.18)	0.03	0.15	0.22<br/> (0.24–0.19)	0.02	0.10
LCC	0.11<br/> (0.12–0.07)	0.02	0.19	0.15<br/> (0.19–0.12)	0.02	0.14
LRPG	0.13<br/> (0.18–0.10)	0.03	0.25	0.15<br/> (0.16–0.12)	0.02	0.14
LLPG	0.17<br/> (0.20–0.13)	0.03	0.19	0.23<br/> (0.28–0.20)	0.03	0.14
LsupG	0.14<br/> (0.17–0.11)	0.03	0.23	0.12<br/> (0.15–0.10)	0.02	0.18
LsubG	0.11<br/> (0.13–0.09)	0.01	0.10	0.12<br/> (0.14–0.09)	0.02	0.18
LPsupC	0.18<br/> (0.24–0.11)	0.05	0.30	0.29<br/> (0.39–0.21)	0.07	0.26
LPsubC	0.07<br/> (0.09–0.06)	0.01	0.15	0.10<br/> (0.13–0.05)	0.03	0.32
RPG	0.40<br/> (0.49–0.38)	0.05	0.13	0.51<br/> (0.58–0.46)	0.05	0.10

SD_N_, Unbiased estimate for Standard Deviation, CV, Coefficient of Variation.

##### Remarks.

The only available information on the anatomy of this species in the literature corresponded to populations from Foux à Draguignan (figure 2, 4, 7, 9 in Boeters 1970 and figure 2 by M. Bodon in Girardi 2009) and Source du Fauvery in Pontevès (figure 1 by M. Bodon in Girardi 2009). The specimens examined from Source d’Argens (Brue-Aurillac) are similar in shell and gastric complex shapes to specimens from Source du Fauvery though they more resemble specimens from the Foux à Draguignan in terms of pallial oviduct shape and number of gill filaments. However, other important diagnostic characters such as the shape of the penis and bursa copulatrix as well as seminal receptacle shape and its position on the renal oviduct are similar in the three populations. Based on these comparisons we conclude that specimens of the three localities belong to the same taxonomic unit with some inter-population variability shown.

Comparingshell sizes among the *Pseudamnicola (Corrosella)* species from the northern half of Iberian Peninsula, the shells of *Pseudamnicola (Corrosella) astieri* are larger (2.5–3.5 mm) than those of *Pseudamnicola (Corrosella) hauffei* sp. n. (2.20–2.90 mm) (see statistically significant differences in shell measurements in Table 2) and *Pseudamnicola (Corrosella) hinzi* Boeters, 1986 (2.2–2.7 mm, Boeters 1986) yet similar in size to those of *Pseudamnicola (Corrosella) navasiana* (Fagot, 1907) (3.0–3.5 mm, Boeters 1988). The only two shell variables resulting no significant between *Pseudamnicola (Corrosella) astieri* and *Pseudamnicola (Corrosella) hauffei* sp. n. were the rate SL/SW and NSW. That means that both species share the same ovate-conic shape and around 4 spire whorls, which are common characteristics among all *Pseudamnicola (Corrosella)* species. Anatomically, *Pseudamnicola (Corrosella) astieri* bears a similar or higher number of gill filaments (about 17–18) than *Pseudamnicola (Corrosella) hinzi* (16–17, Boeters 1986), *Pseudamnicola (Corrosella) navasiana* (15–16, Boeters 1988) and *Pseudamnicola (Corrosella) hauffei* sp. n. (about 15). The penis in *Pseudamnicola (Corrosella) astieri* is narrower and more slender than in *Pseudamnicola (Corrosella) hauffei* sp. n. and *Pseudamnicola (Corrosella) navasiana*, although it is wider and longer than in *Pseudamnicola (Corrosella) hinzi*. The copulatory organ is pigmented in its distal region in all four species, but the pigmentation patch is larger in *Pseudamnicola (Corrosella) astieri*. Although the bursa copulatrix is usually elongate or pyriform shaped among (*Pseudamnicola*) *Corrosella* species, it is U-shaped and folded in *Pseudamnicola (Corrosella) astieri* whereas it is J-shaped in *Pseudamnicola (Corrosella) hauffei* sp. n., *Pseudamnicola (Corrosella) hinzi* and *Pseudamnicola (Corrosella) navasiana*. A small seminal receptacle (around 0.15 mm) is a character common to all four species.

#### Pseudamnicola (Corrosella) hauffei sp. n.

urn:lsid:zoobank.org:act:4DC6D03C-09B8-4E52-924C-39D6B01BBF42

http://species-id.net/wiki/Pseudamnicola_hauffei

##### Type locality.

Los Nogales spring, Benafer, Castellón, Spain, 30°55.80'N, 0°34.34'W.

##### Type material.

**Figure 5.**
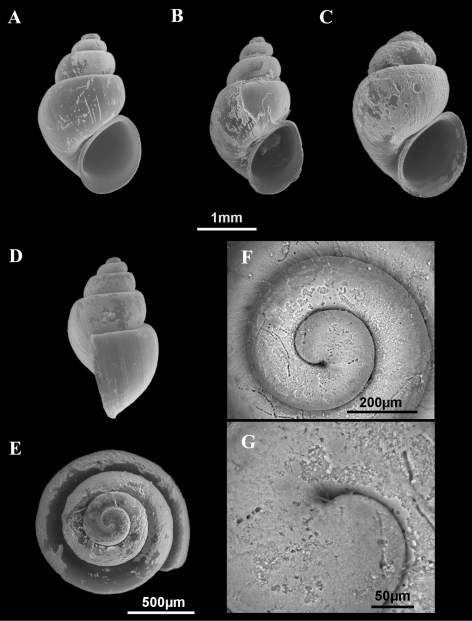
Shells of *Pseudamnicola (Corrosella) hauffei* sp. n. **A, D–G** Shells from Nogales spring, Benafer, Castellón, Spain **B** Shell from San Miguel spring, Viver, Castellón, Spain **C** Shell from Agadín spring, Benafer, Castellón, Spain **E–G** Protoconch and microsculpture.

**Figure 6.**
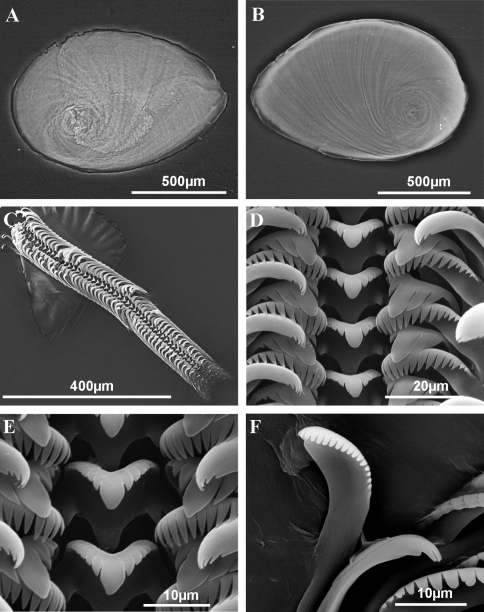
Operculum and radula of *Pseudamnicola (Corrosella) hauffei* sp. n. from Nogales spring, Benafer, Castellón, Spain **A** Internal side of the operculum **B** External side of the operculum **C** Radula **D** Rows of teeth of the radula **E** Central tooth **F** External marginal teeth.

**Figure 7.**
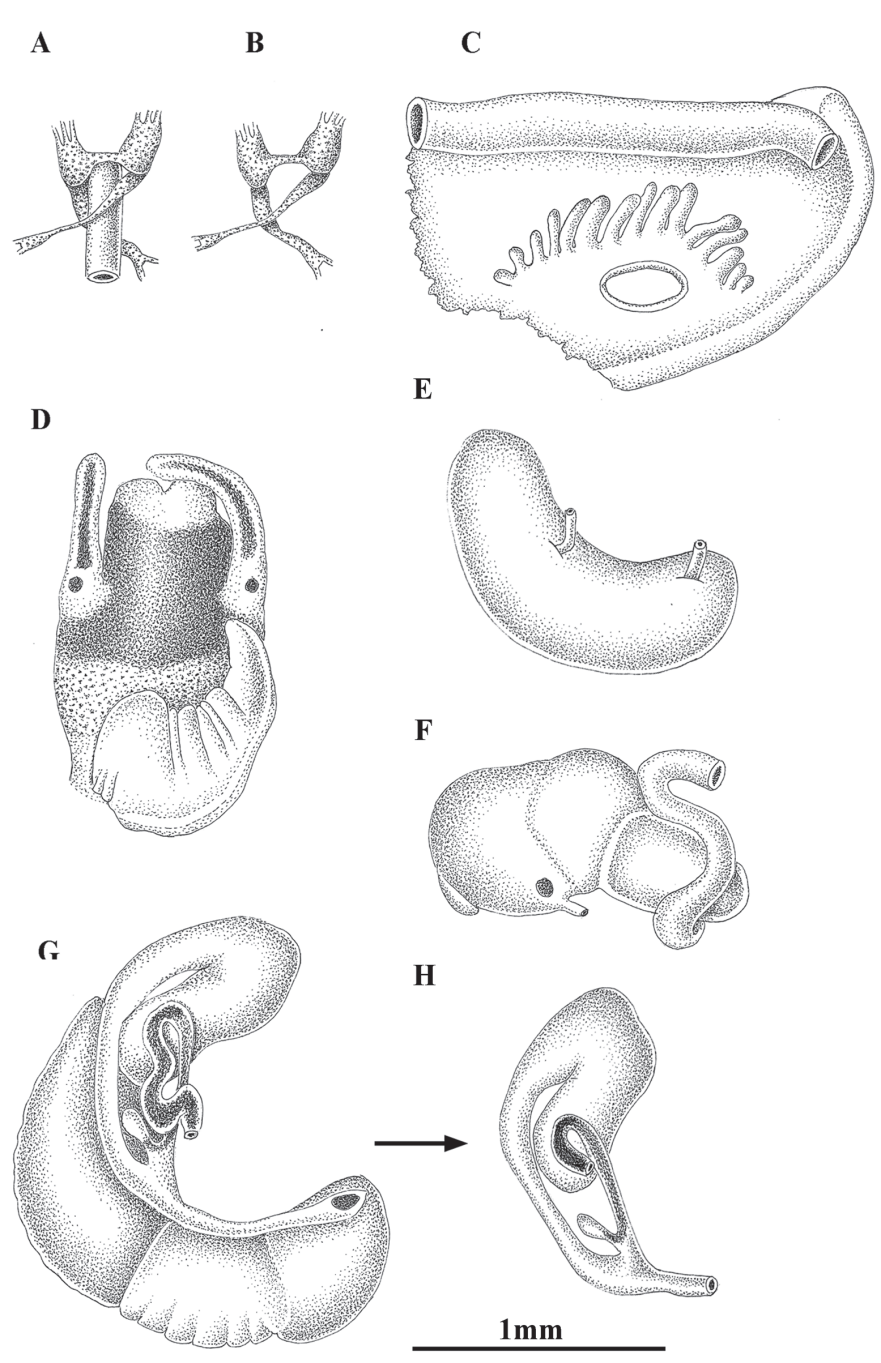
Anatomy of *Pseudamnicola (Corrosella) hauffei* sp. n. from Nogales spring, Benafer, Castellón, Spain. **A,** **B** Partial nervous system **C** Ctenidium and osphradium **D** Head of a male and penis **E** Prostate gland **F** Stomach **G** Female genitalia **H** Bursa copulatrix and seminal receptacle.

Holotype MNCN 15.05/60026a (SEM preparation, Figure 5A) and paratypes (Figures 5D–G, 6, 7) MNCN 15.05/60026b (SEM preparation, Figures 5D–G, 6, and 70° ethanol, Figure 7) and MNCN/ADN 54952–54969 (frozen material and 70° ethanol), D.D. & C.N., 19 March 2009; MNCN 15.05/60027 (70° ethanol), 26 May 1998, B.A.

##### Material examined.

Four males and four females from type locality were examined for anatomical study. In addition, some populations from provinces of Castellón and Valencia (Spain) were also found and studied, dissecting likewise two males and two females from each for their identification.

##### Localities.

Los Nogales spring, Benafer, Castellón, Spain (type locality), 30°55.80'N, 0°34.34'W, B.A., 26 May 1998, MNCN 15.05/60027 (70° ethanol); D.D. & C.N., 19 March 2009, MNCN 15.05/60026 (70° ethanol and ESEM preparation, Figures 5A, D–G) and MNCN/ADN 54952–54969 (frozen material); Agadín spring, Benafer, Castellón, Spain, 39°56.38'N, 0°34.54'W, D.D. & C.N., 19 March 2009, MNCN 15.05/60028 (70° ethanol and ESEM preparation, Figure 5C) and MNCN/ADN 54970–54974 (frozen material); irrigation ditch in Navajas, Castellón, Spain, 39°52.09'N, 0°30.37'W, R.A., D.M. & J.M.R 7 March 1990, MNCN 15.05/60029 (70° ethanol); Curso spring, Navajas, Castellón, Spain, 39°52.43'N, 0°30'W, B.A., 25 May 1998, MNCN 15.05/60030 (70° ethanol) and MNCN/ADN 54975–54989 (frozen material); La Peña spring, Navajas, Castellón, Spain, 39°52.77'N, 0°30.03'W, R.A., D.M. & J.M.R, 7 March 1990, MNCN 15.05/60031 (70° ethanol); La Esperanza spring, Navajas, Castellón, Spain, 39°52.19'N, 0°30.43'W, R.A., D.M. & J.M.R, 7 March 1990, MNCN 15.05/60032 (70° ethanol); Del Prado spring, Viver, Castellón, Spain, 39°56.23'N, 0°36.81'W, D.D. & C.N., 19 March 2009, MNCN 15.05/60033 (70° ethanol) and MNCN/ADN 54990–54992 (frozen material); San Miguel spring, Viver, Castellón, Spain, 39°55.68'N, 0°36.64'W, B.A., 25 May 1998, MNCN 15.05/60034 (70° ethanol); D.D. & C.N., 19 March 2009, MNCN 15.05/60035 (70° ethanol and ESEM preparation, Figure 5B) and MNCN/ADN 54997–54999 (70° ethanol); San Miguel ditch, Viver, Castellón, Spain, 39°55.68'N, 0°36.64'W, D.D. & C.N., 19 March 2009, MNCN 15.05/60036 (70° ethanol) and MNCN/ADN 54993–54996 (frozen material); Font Nova, Benifaió, Valencia, Spain, 39°0.55'N, 0°5.87'W, B.A., 26 May 1998, MNCN 15.05/60037 (70° ethanol); Cortés de Pallás, Valencia, Spain, 39°14.61'N, 0°26.01'W, B.A., 26 May 1998, MNCN 15.05/60038 (70° ethanol).

##### Material examined for morphometry.

Shell, anatomical, operculum and radular measurements (Tables 2–7) were made on specimens from the type locality, Los Nogales spring in Benafer, Castellón.

##### Etymology.

Dedicated to the malacologist and ecologist Torsten Hauffe, for his help and support during the stay of the first author in Germany.

##### Diagnosis.

Shell yellowish with body whorl occupying 2/3 shell length; umbilicus slightly visible; protoconch microsculpture grooved; central radular tooth formula 5-C-5; style sac protruding below non-pigmented intestine; elongate bursa copulatrix J-shaped; renal oviduct pigmented until seminal receptacle, which has a pigmented short duct; penis triangular with a wide base attached to central area of head; nervous system brown pigmented with supraoesophageal connective about three times longer than suboesophageal.

##### Description.

Shellovate-conic (Figure 5A–C), yellowish periostracum with 4–4.5 spire whorls, height around 2.0–3.0 mm (Table 2); protoconch approximately 450 µm wide with 1.5 whorls and a nucleus around 200 µm long (Figure 5E, F); protoconch microsculpture grooved (Figure 5G); body whorl about 2/3 total length; whorls convex with deep suture; peristome frontal, complete, oval, with thick inner lip partly hiding umbilicus; outer peristome simple, straight (Figure 5D).

Operculum corneous, yellowish, thin, pliable, ellipsoidal, paucispiral, with nucleus submarginal (Figure 6A, B; Table 3); oval muscle attachment near nucleus.

Radula with around 50 rows of teeth, medium in size (25% total shell length) (Figure 6C, Table 4); central tooth with a tongue-shaped median cusp and five lateral cusps, slightly sharpening towards central one (Figure 6D, E); lateral teeth with a long tongue-shaped median cusp and three tapered laterals; inner and outer marginal teeth bear 15 and 19 sharp cusps respectively (Figures 6D, F).

*Pigmentation and anatomy*: Head intensely brown pigmented from snout to neck (Figure 7D); pigment on neck clearer than on head; brown band of pigment also on tentacles, but not on ocular lobes; snout as long as wide, with medial lobation; foot intermediate length, pigmented on dorsal region. Ctenidium in the anterior region of pallial cavity with about 15 gill filaments; osphradium ellipsoidal under central gill filaments (Figure 7C, Table 5). Stomach slightly longer than wide (Figure 7F); style sac barely shorter than stomach, protruding below intestine (Table 5).

**Figure 8.**
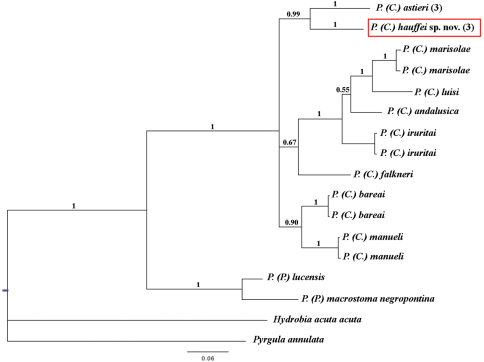
Bayesian 50% majority rule consensus tree inferred employing COI mitochondrial gene partial sequence. The numbers above branches represent Bayesian posterior probabilities. The numbers between brackets symbolize specimens with identical haplotypes. Scale bar: expected changed per site.

Female genitalia with a pallial oviduct about four times longer than wide (Figure 7G; Table 6); capsule gland slightly longer than albumen gland and denser in posterior region; genital aperture in the anterior extreme of pallial oviduct; elongate bursa copulatrix, J-shaped folded with a duct less than 50% bursa length; renal oviduct scarcely pigmented from the insertion point of bursal duct to where it begins to fold and black pigmented, making two or three loops; elongate seminal receptacle with pigmented short duct (Figure 7H) joining renal oviduct slightly above the point where the bursal duct joins the renal oviduct.

Male genitalia bearing a bean-like prostate gland about three times longer than wide (Figure 7E, Table 6); penis triangular with a wide base attached to central area of head with some folds in middle section and a narrow patch of black pigment on distal surface (Figure 7D); vas deferens uncoiled in penis running straight close to the external margin.

Nervous system brown pigmented, but ganglia darker than connectives and commissures; cerebral ganglia equal in size; supraoesophageal and suboesophageal ganglia similar in shape and size; supraoesophageal connective around three times longer than suboesophageal (Figure 7A, B; Table 7). Mean RPG ratio 0.51 (elongated).

##### Remarks.

Some of the localities where this species was found were cited by Gasull (1981) incorrectly as inhabited by *Pseudamnicola (Corrosella) astieri*. Both species show marked differences such as: 1) *Pseudamnicola (Corrosella) astieri* has a longer shell, longer spire (SL-LBW) (Table 2) and the protoconch microsculpture is more granulated than in *Pseudamnicola (Corrosella) hauffei* sp. n. Moreover, the inner lip of the shell aperture in *Pseudamnicola (Corrosella) hauffei* sp. n. is thicker than in *Pseudamnicola (Corrosella) astieri* and partly hides the umbilicus; 2) central radular tooth with seven lateral cusps in *Pseudamnicola (Corrosella) astieri*, five in *Pseudamnicola (Corrosella) hauffei* sp. n. (Table 4); 3) style sac surrounded by black pigmented intestine in *Pseudamnicola (Corrosella) astieri* yet lacks pigment and protrudes under the intestine in *Pseudamnicola (Corrosella) hauffei* sp. n. (Figures 4F and 7F); 4) bursa copulatrix J-shaped and seminal receptacle with a short duct in *Pseudamnicola (Corrosella) hauffei* sp. n., while bursa copulatrix is U-shaped and seminal receptacle is shorter and lacks a duct in *Pseudamnicola (Corrosella) astieri* (Figures 4H and 7H); 5) penis triangular with a wide base in *Pseudamnicola (Corrosella) hauffei* sp. n. and slender in *Pseudamnicola (Corrosella) astieri* (Figures 4D and 7D); 6) nervous system elongated (RPG= 0.51) in *Pseudamnicola (Corrosella) hauffei* sp. n. yet moderately concentrated (RPG= 0.42) in *Pseudamnicola (Corrosella) astieri*.

Compared to the other *Pseudamnicola (Corrosella)* species living in nearby areas, *Pseudamnicola (Corrosella) hinzi* and *Pseudamnicola (Corrosella) navasiana*, *Pseudamnicola (Corrosella) hauffei* sp. n. has a shorter and more ovate shell shape, a longer bursa copulatrix, bursa duct and seminal receptacle, and a more triangular wider-based penis.

## Molecular analysis

The data set analysed included data for 11 *Pseudamnicola* species and 658 characters of the COI gene. New sequences for both species were deposited in Genbank under accession numbers JQ067672 – JQ067677, while the rest of the sequences were obtained from this same database (see Table 1). *Hydrobia acuta acuta* (Draparnaud, 1805) and *Pyrgula annulata* (Linnæus, 1758) were used as outgroups.

*Pseudamnicola (Corrosella) hauffei* sp. n. differed 7.44% with respect to *Pseudamnicola (Corrosella) astieri* specimens and moreover, both were clustered as sister species (Table 8 and Figure 8). Through Bayesian analysis, the subgenus *Corrosella* was found to be well supported and divided into three clades, whose phylogenetic relationships are still unclear. The clades comprising *Pseudamnicola (Corrosella) hauffei* sp. n. and *Pseudamnicola (Corrosella) astieri*, or *Pseudamnicola (Corrosella) manueli* and *Pseudamnicola (Corrosella) bareai* were well supported (posterior probabilities over 0.90). However the clade including *Pseudamnicola (Corrosella) marisolae*, *Pseudamnicola (Corrosella) luisi*, *Pseudamnicola (Corrosella) andalusica*, *Pseudamnicola (Corrosella) iruritai* and *Pseudamnicola (Corrosella) falkneri* was not well supported.

**Table 8.** Genetic divergence matrix for the species examined based on the COI gene sequence.

	**1**	**2**	**3**	**4**	**5**	**6**	**7**	**8**	**9**	**10**	**11**	**12**	**13**
1. *Pseudamnicola (Corrosella) astieri*	-												
2. *Pseudamnicola (Corrosella) hauffei* sp. n.	7.44	-											
3. *Pseudamnicola (Corrosella) marisolae*	10.26	10.41	-										
4. *Pseudamnicola (Corrosella) luisi*	10.79	12.16	6.00	-									
5. *Pseudamnicola (Corrosella) iruritai*	8.13	9.42	6.38	6.69	-								
6. *Pseudamnicola (Corrosella) andalusica*	9.11	10.64	6.46	6.54	5.39	-							
7. *Pseudamnicola (Corrosella) falkneri*	8.97	8.81	9.19	10.33	7.83	8.05	-						
8. *Pseudamnicola (Corrosella) bareai*	7.86	7.65	9.14	9.59	7.87	8.50	8.42	-					
9. *Pseudamnicola (Corrosella) manueli*	8.73	8.74	9.65	11.15	8.81	9.03	7.67	5.46	-				
10. *Pseudamnicola (Pseudamnicola) lucensis*	15.06	14.78	15.15	15.21	13.69	14.31	13.66	12.90	14.22	-			
11. *Pseudamnicola (Pseudamnicola) macrostoma negropontina*	14.45	14.47	14.85	14.89	14.16	14.32	13.67	14.42	14.54	7.68	-		
12. *Hydrobia acuta acuta*	17.91	19.49	17.60	17.26	15.78	16.35	16.62	17.12	18.29	18.65	18.97	-	
13. *Pyrgula annulata*	16.67	17.44	17.55	16.67	14.99	16.82	17.58	16.09	17.05	16.90	18.14	17.38	-

## Conclusions

Based on this wide morphological study and our molecular data, we were able to delimit both species and clearly rule out the hypothesis of the presence of *Pseudamnicola (Corrosella) astieri* in the Iberian Peninsula, identifying it as an endemism of the Alpes-Maritimes and Var departments of France, as proposed by Falkner in 2002. Consequently, the Iberian populations formerly cited as *Pseudamnicola (Corrosella) astieri* in Castellón province (Gasull 1981, Vidal-Abarca and Suárez 1985) actually correspond to the new species *Pseudamnicola (Corrosella) hauffei* sp. n. Morphologically, the most diagnostic characters are provided by shell habitus, central radular tooth, male and female genital systems and the RPG ratio. In effect, a considerable difference between the two species was detected in nervous system condensation (RPG ratio). In addition, *Pseudamnicola (Corrosella) hauffei* sp. n. has more elongated connectives, which is considered a primitive state (Fretter and Graham 1962).

Through a phylogenetic approach based on partial sequence data for the COI gene provided in GenBank for other *Pseudamnicola (Corrosella)* species, we were able to estimate a mean genetic divergence of about 8% (5.39 to 11.15%) (Delicado et al. 2012). In comparison, our preliminary molecular data for the same gene sequence indicate a genetic divergence of 7.4% between *Pseudamnicola (Corrosella) astieri* and *Pseudamnicola (Corrosella) hauffei* sp. n., suggesting the two taxa are in fact different taxonomic entities.

Besides clarifying the taxonomic status of these two species and their phylogenetic relationship as sister species, our findings point to a greater diversity of *Pseudamnicola (Corrosella)* than previously thought, with implications for the protection of this poorly known group of molluscs. Indeed, their fragile ecosystems susceptible to the effects of human activities, altered water regimes, pollution, etc. means that most of these hydrobiid species are seriously threatened or even endangered (see Hydrobiidae spp. by Arconada et al. in Verdú and Galante 2006). Moreover, the fact that both taxonomic entities, *Pseudamnicola (Corrosella) astieri* and *Pseudamnicola (Corrosella) hauffei* sp. n. are endemisms inhabiting restricted areas of France and Spain respectively instead of belonging to a single species with a large distribution area, suggests that they should be assessed for inclusion in the Red Lists of both countries and conservation measures should be taken to protect their fragile habitats.

## Supplementary Material

XML Treatment for Pseudamnicola (Corrosella) astieri

XML Treatment for Pseudamnicola (Corrosella) hauffei
